# Prognostic biomarkers for predicting decompensation in alcoholic and non-alcoholic patients with compensated cirrhosis: a systematic review and meta-analysis

**DOI:** 10.3389/fmed.2025.1650124

**Published:** 2025-09-03

**Authors:** Kristina Baktikulova, Saulesh Kurmangaliyeva, Kairat Kurmangaliyev, Konstantin Tissin, Nadiar M. Mussin, Amin Tamadon

**Affiliations:** ^1^Department of Microbiology, Virology and Immunology, West Kazakhstan Marat Ospanov Medical University, Aktobe, Kazakhstan; ^2^Department of Surgery No. 2, West Kazakhstan Marat Ospanov Medical University, Aktobe, Kazakhstan; ^3^Department of Natural Sciences, West Kazakhstan Marat Ospanov Medical University, Aktobe, Kazakhstan

**Keywords:** biomarkers, disease, progression liver, cirrhosis prognosis, humans

## Abstract

**Background:**

Hepatic decompensation is a critical turning point in the progression of compensated cirrhosis, with distinct pathophysiological trajectories in alcoholic and non-alcoholic etiologies. This systematic review and meta-analysis evaluates prognostic biomarkers for predicting decompensation in patients with compensated cirrhosis, emphasizing differences between alcoholic and non-alcoholic liver disease.

**Methods:**

Following PRISMA 2020 guidelines, we systematically searched PubMed, Scopus, and Web of Science for peer-reviewed studies (up to April 2025) reporting hazard ratios (HRs) and 95% confidence intervals for biomarkers predicting decompensation in adults with compensated cirrhosis. Eligible studies included observational cohorts and control arms of RCTs, stratified by etiology (alcoholic vs. non-alcoholic). Data were pooled using random-effects models, with heterogeneity assessed via *I*^2^ and Cochrane Q tests. Subgroup analyses explored biomarker performance by etiology and type (inflammatory, functional, and structural).

**Results:**

From 691 records, 66 studies (Among these, 955 patients (2.6%) were alcoholic and 36,108 (97.4%) non-alcoholic, totaling 37,063 participants) were included. In non-alcoholic cirrhosis, structural biomarkers like portal vein diameter (HR = 7.39 [4.90, 11.15]) and spleen size (HR = 5.79 [2.00, 16.80]) were strong predictors, alongside functional markers such as bilirubin (HR = 4.27 [2.93, 6.22]) and MELD score (HR = 1.13 [1.07, 1.20]). In alcoholic cirrhosis, inflammatory biomarkers, particularly extracellular vesicles (HR = 5.09 [2.01, 12.86]) and keratin-18 (HR = 1.77 [1.14, 2.75]), showed superior predictive value. Interleukin-6 was predictive across both etiologies (HR = 1.31 [1.00, 1.71]). Heterogeneity was substantial (*I*^2^ > 50%) for most biomarkers, reflecting population and methodological variability. Publication bias was low based on funnel plots and Egger’s test.

**Conclusion:**

Etiology-specific biomarkers enhance prognostic accuracy in compensated cirrhosis. Structural and functional markers dominate in non-alcoholic cirrhosis, while inflammatory biomarkers are more predictive in alcoholic cirrhosis. Integrating these into personalized risk models could improve clinical management, though prospective validation is needed.

**Systematic Review Registration:**

https://www.crd.york.ac.uk/PROSPERO/view/CRD420251076849

## 1 Introduction

Cirrhosis represents the final common pathway of progressive liver fibrosis and remains a significant global health burden, accounting for over one million deaths annually worldwide (1). Liver stiffness measurement (LSM) via transient elastography has also emerged as a non-invasive surrogate for portal hypertension and hepatic decompensation risk. Importantly, the etiology of cirrhosis—whether alcoholic liver disease (ALD) or metabolic dysfunction-associated steatotic liver disease (MASLD, formerly NAFLD)—may influence both disease trajectory and biomarker performance (2). For instance, alcoholic cirrhosis is often more inflammatory and rapidly progressive, while MASLD-related cirrhosis may involve more gradual fibrotic remodeling influenced by insulin resistance, dyslipidemia, and gut-derived signals (3, 4).

If we consider alcoholic cirrhosis arises as the final stage of ALD, which follows a progressive pathological sequence: steatosis, steatohepatitis, fibrosis, and cirrhosis (5). Chronic and excessive alcohol consumption (>30 g/day in men, >20 g/day in women) induces hepatic injury through multiple converging mechanisms (6). Ethanol metabolism generates toxic intermediates such as acetaldehyde and reactive oxygen species, which cause mitochondrial dysfunction, lipid peroxidation, and DNA damage in hepatocytes (7). Concurrently, alcohol disrupts gut barrier integrity, increasing lipopolysaccharide translocation from the gut microbiota into the portal circulation (8). This promotes Kupffer cell activation and chronic hepatic inflammation via toll-like receptor 4 signaling (9).

Moreover, alcohol impairs liver regeneration and augments fibrogenesis by stimulating hepatic stellate cells, resulting in progressive deposition of extracellular matrix proteins (10). Genetic and epigenetic factors (e.g., PNPLA3 variants), nutritional deficiencies, and coexisting hepatitis viruses further accelerate fibrosis (11). Importantly, compared to metabolic cirrhosis, alcoholic cirrhosis often progresses more rapidly, and the risk of decompensation is heightened by ongoing alcohol intake, malnutrition, and systemic inflammation (12).

Understanding these unique pathogenic features is crucial when interpreting biomarker dynamics in ALD. Inflammatory markers, macrophage activation indices, and gut-derived metabolites may have greater prognostic significance in alcoholic cirrhosis, offering opportunities for etiology-specific risk prediction (13, 14). Yet, few studies have directly compared biomarker utility across these etiologies in the context of decompensation prediction. This systematic review and meta-analysis aim to synthesize current evidence on prognostic biomarkers that predict hepatic decompensation in patients with compensated cirrhosis, with a specific focus on differences between alcoholic and non-alcoholic etiologies. We aimed to address the question: In adults with compensated cirrhosis (Population), which biomarkers (Exposure) predict hepatic decompensation (Outcome), and how does their prognostic performance differ between alcoholic and non-alcoholic etiologies (Comparison)? Through critical appraisal of study quality and meta-analytical pooling of predictive metrics, we seek to clarify the clinical utility and limitations of these biomarkers in guiding individualized management.

## 2 Materials and methods

This meta-analysis was conducted according to the recommendations outlined in the PRISMA 2020 guidelines. These standards were applied throughout all stages of the study, including study design, data collection, statistical analysis, and interpretation of results. The study protocol was registered in the PROSPERO database under the registration number CRD420251076849. Institutional Review Board approval was not required, as this was a meta-analysis.

### 2.1 Literature search

To identify studies relevant to the objective of this meta-analysis, a systematic search was conducted in the PubMed, Web of Science, and Scopus databases using comprehensive search terms detailed in Table 1. The search was restricted to studies involving human subjects, specifically focusing on full-text articles published in peer-reviewed journals in English. Since gray literature, such as conference abstracts and unpublished data, typically lacks peer review, its inclusion could compromise the reliability of the meta-analysis findings; therefore, such sources were excluded. Additionally, the reference lists of relevant original and review articles were manually screened to identify other potentially eligible studies. The literature search covered the period from the inception of each database through April 2025. No additional sources, such as trial registries or conference proceedings, were searched due to the focus on peer-reviewed full-text articles. The last search was conducted on April 30, 2025, prior to data analysis and manuscript drafting in July 2025.

**Table 1:** Search strategy for the meta-analysis of prognostic biomarkers predicting decompensation in alcoholic and non-alcoholic compensated cirrhosis across PubMed, Scopus, and Web of Science.

Code	Search query
#1	TITLE-ABS-KEY (“compensated cirrhosis” OR “compensated liver cirrhosis”)
#2	TITLE-ABS-KEY (“biomarker” OR “prognostic marker” OR “serum marker”)
#3	TITLE-ABS-KEY (“decompensation” OR “ascites” OR “hepatic encephalopathy” OR “variceal bleeding”)
#4	TITLE-ABS-KEY (“hazard ratio” OR “risk factor” OR “prognosis”)
#5	#1 AND #2 AND #3 AND #4

### 2.2 Inclusion and exclusion criteria

This review included studies that met specific eligibility criteria regarding population, study design, exposure, outcome, and data reporting. Compensated cirrhosis was defined as the absence of clinically apparent complications such as ascites, variceal hemorrhage, or hepatic encephalopathy, based on the Baveno VII consensus, which incorporates criteria such as liver stiffness measurement <15 kPa and platelet count >150 × 10^9^/L as non-invasive surrogates for portal hypertension. These standards enable early identification and stratification of patients prior to clinical decompensation. The target population comprised adults aged 18 years or older diagnosed with compensated liver cirrhosis, defined according to the Baveno VII criteria or other validated clinical standards. Studies were eligible if they included, or provided stratified data for, patients with both alcoholic and non-alcoholic etiologies of cirrhosis (15). Acceptable study designs were observational cohort studies—either prospective or retrospective—as well as the control arms of randomized controlled trials (RCTs). Included studies were required to assess clinical, biochemical, physiological, or imaging-derived biomarkers as potential predictors of hepatic decompensation. The primary outcome of interest was the occurrence of hepatic decompensation events, including ascites, variceal hemorrhage, or hepatic encephalopathy. To ensure sufficient quantitative data for synthesis, only studies that reported hazard ratios (HRs) with complete 95% confidence intervals (CIs) (including both lower and upper bounds) for predictors of hepatic decompensation were considered.

Exclusion criteria were also clearly defined. Studies were excluded if they involved non-human or preclinical research, such as experimental work conducted in animal models or *in vitro* settings. Ineligible study designs included cross-sectional studies, case reports, case series, editorials, letters to the editor, narrative reviews, systematic reviews, and meta-analyses. Additionally, studies that exclusively focused on patients with decompensated cirrhosis, without inclusion of individuals in the compensated phase, were excluded. Finally, studies restricted solely to patients with hepatocellular carcinoma, without addressing a broader cirrhosis population, were also excluded from the analysis. Studies were grouped for synthesis based on biomarker type (inflammatory, functional, structural) and cirrhosis etiology (alcoholic vs. non-alcoholic) to explore etiology-specific prognostic performance. Events such as hepatorenal syndrome, acute liver failure, and coagulopathy-induced bleeding were variably reported and not included as primary decompensation outcomes in this review to maintain consistency across the included studies.

### 2.3 Study quality evaluation and data extraction

The selection and quality assessment of included studies were conducted according to a predefined protocol. Two independent reviewers (KK and SK) systematically searched PubMed, Scopus, and Web of Science for studies investigating biomarkers as predictors of decompensation in alcohol- and non-alcohol-related liver cirrhosis. During the initial screening phase, titles and abstracts were evaluated independently by both reviewers, and duplicate entries were manually removed. Screening and duplicate removal were performed manually. Full-text review proceeded only when both reviewers concurred on inclusion eligibility. Discrepancies were resolved through discussion; unresolved cases were adjudicated by a third reviewer (KB) to ensure impartiality. Data extraction was completed independently by both reviewers (SK and KK) in April 2025, ensuring consistency. Extracted information encompassed: General study characteristics: Authors, publication year, country, study design. Participant characteristics: Sample size, age, sex, and liver disease etiology. Biomarker assessment: Type, measurement methods, threshold values, and prevalence of biomarker positivity. Follow-up: Duration and outcomes (e.g., decompensation). Analytical covariates: Variables included in regression models. No attempts were made to contact study investigators for missing or unclear data, as only studies with complete HRs and 95% CIs were included. All reported results compatible with hepatic decompensation (across time points and analyses) were sought. Missing or unclear data were excluded, assuming reported HRs reflected adjusted estimates unless otherwise specified. Risk of bias was assessed using a modified Quality In Prognosis Studies (QUIPS) framework, with two reviewers (KT, KK) independently evaluating each study and discrepancies resolved by a third reviewer (KB).

### 2.4 Statistics

The objective of this meta-analysis was to investigate the prognostic value of traditional and emerging biomarkers for predicting the first episode of hepatic decompensation in patients with compensated cirrhosis of alcoholic and non-alcoholic etiology. Studies were eligible for synthesis if they provided complete HRs and CIs for biomarkers with clinical relevance, selected to avoid overlapping cohorts and ensure comparability across etiologies. For each biomarker, HRs with corresponding 95% CIs were extracted or calculated. When only *p*-values or CIs were reported, standard errors (SEs) were derived manually. Logarithmic transformation was applied to stabilize variance and normalize the distribution of HRs.

Heterogeneity across studies was assessed using the Cochrane Q test and the *I*^2^ statistic. An *I*^2^ value above 50% was considered indicative of substantial heterogeneity (16). To account for between-study variability, a random-effects model was applied.

Sensitivity analyses were conducted by sequentially excluding individual studies to evaluate the robustness of the pooled estimates. Additionally, a predefined subgroup analysis was performed to examine how biomarker type (inflammatory, functional, or structural) and cirrhosis etiology (alcoholic vs. non-alcoholic) influenced predictive performance. This stratification enabled comparisons between inflammatory markers [neutrophil-to-lymphocyte ratio (NLR), platelet-to-lymphocyte ratio (PLR), and interleukin-6 (IL-6)], functional markers (e.g., albumin and bilirubin), and structural indicators [spleen size, portal vein diameter (PVD)]. Etiology-based subgrouping allowed for exploration of pathophysiological differences—for instance, inflammatory markers showed greater predictive strength in alcoholic cirrhosis, while structural markers performed better in non-alcoholic cases.

Additional modifying factors, such as biomarker threshold values and follow-up duration, were also evaluated. Differences between univariable and multivariable model outcomes were analyzed to assess the independent prognostic contribution of each biomarker. All statistical analyses were conducted using Review Manager (RevMan) version 5.1 (Cochrane Collaboration, Oxford, United Kingdom). A *p*-value less than 0.05 was considered statistically significant.

### 2.5 Reporting bias assessment

Publication bias was assessed using funnel plots for syntheses with ≥10 studies per biomarker, supplemented by Egger’s test to detect asymmetry. Selective reporting was evaluated by comparing reported outcomes to study protocols, where available.

## 3 Results

### 3.1 Study inclusion

**Figure 1:**
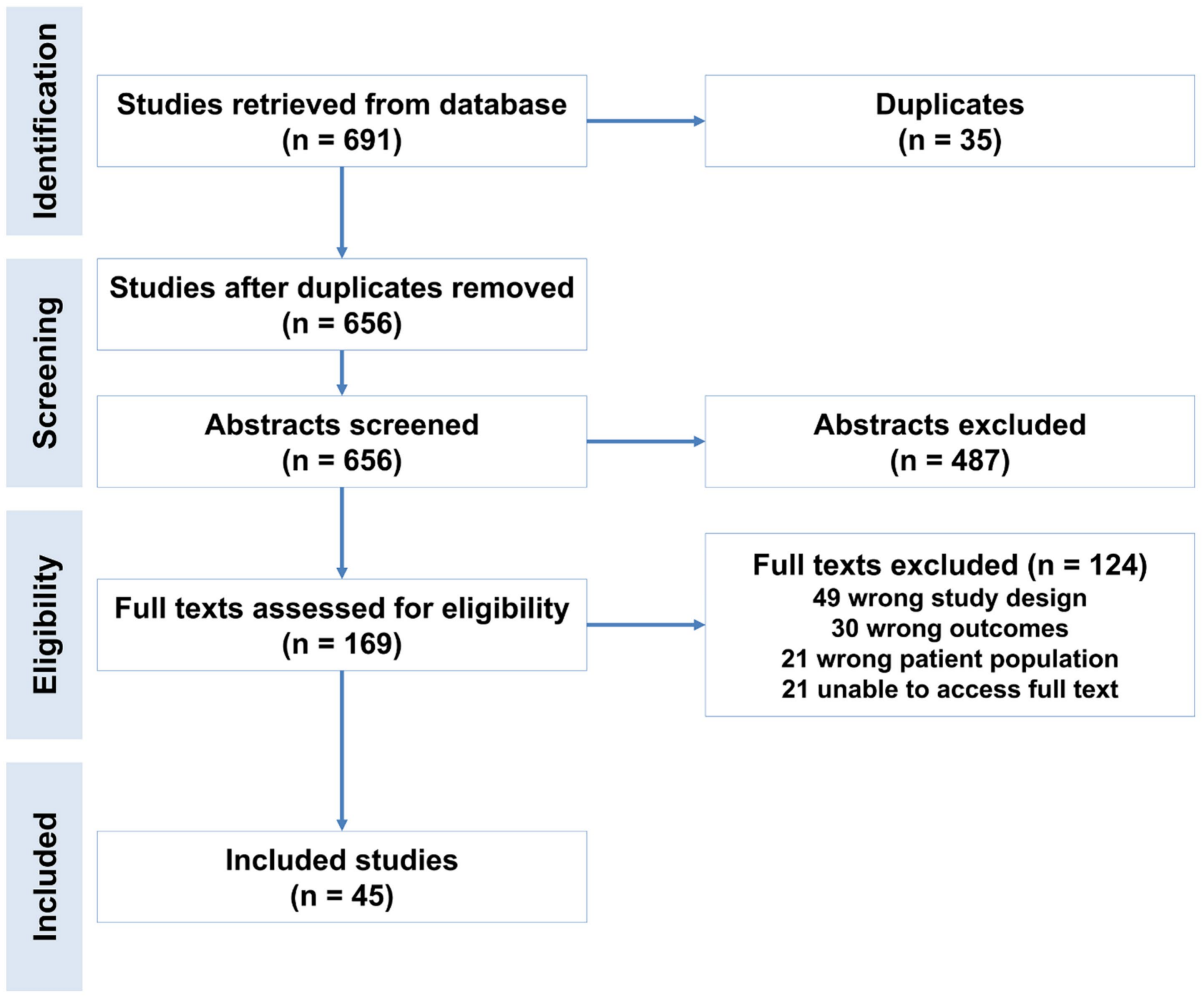
Flowchart of study selection for the meta-analysis of prognostic biomarkers predicting decompensation in alcoholic and non-alcoholic compensated cirrhosis.

The study selection process is summarized in Figure 1 (PRISMA flowchart). A total of 691 records were identified through systematic searches of three databases (e.g., PubMed, Scopus, and Web of Science). After removing 35 duplicate records, 656 unique studies underwent title/abstract screening. Of these, 487 studies were excluded due to irrelevance to the meta-analysis objectives (e.g., non-human studies, reviews, or unrelated outcomes).

The remaining 169 studies advanced to full-text review, conducted independently by two reviewers. At this stage, 66 studies were excluded for the following reasons: Ineligible population: Studies not focused on compensated cirrhosis. Outcome mismatch: Lack of data on biomarker-predicted decompensation. Study design: Case reports, non-English articles, or insufficient statistical data. Ultimately, 66 studies met inclusion criteria and were included in the meta-analysis (17).

### 3.2 Data extraction details

For each eligible study, detailed information was systematically extracted to ensure consistency and enable meaningful comparisons across studies. This included the author(s), year of publication, and study location; the study design and sample size; and key patient characteristics such as age and cirrhosis etiology (alcoholic versus non-alcoholic). Data on the type and category of biomarkers evaluated, measurement methods, and any reported cutoff values were also collected. Clinical outcomes were recorded, specifically the type of hepatic decompensation events observed, follow-up duration, and the number of events. Effect sizes were extracted as HRs or odds ratios (ORs), each accompanied by 95% CIs.

When a study evaluated multiple biomarkers, each was treated as an independent data point in the pooled analysis. Likewise, in studies that included more than one cohort, such as derivation and validation groups, each cohort was analyzed separately if data were available. The literature search and screening process were conducted independently by two reviewers (KT and KK), with duplicates removed automatically. Titles and abstracts were screened independently, and studies advanced to full-text review only when both reviewers agreed on inclusion. A third reviewer (KB) resolved any disagreements and participated in the full-text triage process using the same criteria. The overall study selection process is depicted in Figure 1.

Data extraction was independently performed by KT, KK, and KB. Extracted variables encompassed study design, cirrhosis etiology, follow-up duration, liver disease severity scores, biomarker types, and characteristics. Outcomes related to hepatic decompensation, as well as the statistical methods applied in each study, were also recorded. When HRs and their SEs were not directly provided, they were estimated using the RevMan 5.1 calculator tool. Only biomarkers demonstrating the strongest predictive value were included in the final analysis. These included albumin, platelets, bilirubin, international normalized ratio (INR), model for end-stage liver disease (MELD), hepatic venous pressure gradient (HVPG), liver stiffness, IL-6, M30, M65, keratin-18 (K18), interferon-gamma inducible protein (IEV), and cytokeratin-18 cleaved fragment (CK-18/CPA). In studies that assessed multiple biomarkers, each was analyzed individually, leading to varying numbers of studies contributing to the analysis for each biomarker.

### 3.3 Assessment of study quality

**Figure 2:**
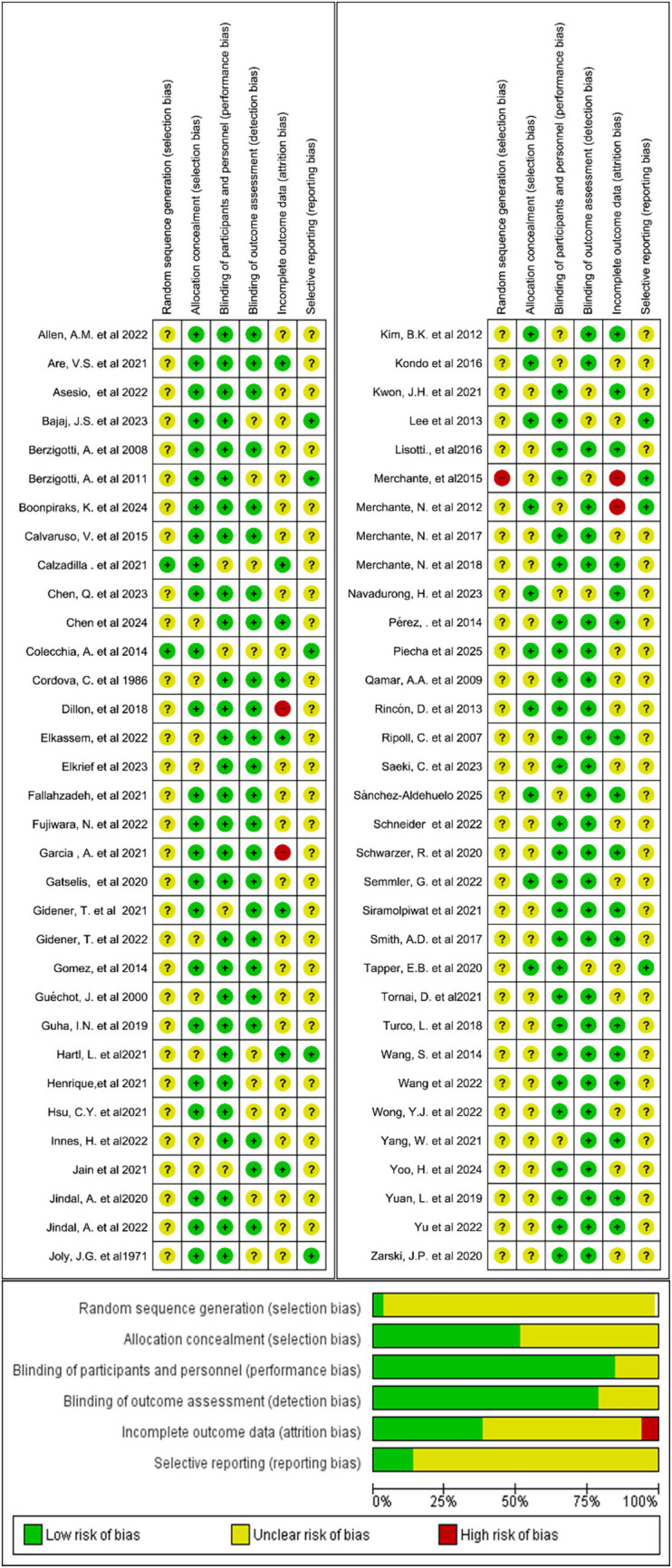
Summary of risk of bias across all included studies for prognostic biomarkers for predicting decompensation in alcoholic and non-alcoholic patients with compensated cirrhosis.

The quality and risk of bias of the included studies were assessed manually using RevMan 5.1. The evaluation encompassed six key domains: study participation, attrition, prognostic factor measurement, outcome measurement, confounding, and statistical analysis. Each study was rated as having a low, moderate, or high risk of bias for each domain and overall. Figure 2 illustrates the proportion of studies classified as having low, unclear, or high risk of bias across seven methodological domains. The majority of studies demonstrated low risk across most domains, while some showed unclear risk, particularly in random sequence generation, allocation concealment, and incomplete outcome data. Notably, no domain was associated with a high risk of bias.

### 3.4 Study characteristics

The meta-analysis encompassed data from a total of 66 studies, including 37,063 patients with compensated liver cirrhosis. Among these, 955 patients (2.6%) were identified as having alcoholic cirrhosis, while the remaining 36,108 patients (97.4%) were classified as having non-alcoholic cirrhosis, which included etiologies such as non-alcoholic fatty liver disease (NAFLD) or non-alcoholic steatohepatitis (NASH), viral hepatitis, and autoimmune liver diseases, as summarized in Table 2. In terms of study design, 28 studies (42.4%) were prospective cohort studies, 30 (45.5%) were retrospective cohort studies, and 8 (12.1%) were RCTs, reflecting a diverse range of methodological approaches across the included literature.

**Table 2:** Frequently studied prognostic biomarkers for hepatic decompensation based on study design and cirrhosis etiology in included studies.

Author, Reference	Year	Patients	Patients with outcome	Biomarker(s)	Design	Etiology
Allen et al. (17)	2022	5,123	5,123	Albumin Bilirubin	Prospective cohort	Non-alcoholic
Are et al. (31)	2021	162	162	ELF score TIMP-1 PIINP hyaluronic acid	RCT	Non-alcoholic
Asesio et al. (22)	2022	455	455	Liver stiffness Albumin Bilirubin Platelets INR	Retrospective cohort	Alcoholic
Berzigotti et al. (32)	2011	161	161	BMI	RCT	Non-alcoholic
Boonpiraks et al. (33)	2024	457	457	Diabetes CP score MELD	Retrospective cohort	Non-alcoholic
Calvaruso et al. (34)	2015	118	118	CPA Albumin Platelets	Prospective cohort	Non-alcoholic
Calzadilla-Bertot et al. (19)	2021	543	543	ABIDE score Albumin Bilirubin Platelets AST/ALT INR	Retrospective cohort	Non-alcoholic
Chen et al. (21)	2024	182	182	Liver stiffness FF Abstinence	Prospective cohort	Non-alcoholic
Chen et al. (35)	2023	688	688	Laminin Collagen IV GGT Platelets	Retrospective cohort	Non-alcoholic
Colecchia et al. (36)	2014	122	92	HVPG Spleen stiffness Platelets MELD score AST/ALT	Prospective cohort	Non-alcoholic
Elkassem et al. (37)	2022	191	191	Liver surface nodularity	Retrospective cohort	Non-alcoholic
Elkrief et al. (24)	2023	500	500	Keratin 18	Prospective cohort	Alcoholic
Fallahzadeh et al. (38)	2021	70	35	Hepquant-SHUNT test	Prospective cohort	Non-alcoholic
Fujiwara et al. (39)	2022	122	122	PLSec VCAM-1 IGFBP-7 gp130 matrilysin IL-6 CCL-21 Angiogenin protein S	Prospective cohort	Non-alcoholic
Garcia et al. (40)	2021	105	105	Serum miR-181b-5p	RCT	Non-alcoholic
Gidener et al. (26)	2021	829	194	Liver stiffness	Retrospective cohort	Non-alcoholic
Gidener et al. (41)	2022	1,269	277	Liver stiffness MELD score CP score	Retrospective cohort	Non-alcoholic
Gomez et al. (42)	2014	402	402	Platelets AST/ALT INR	Prospective cohort	Non-alcoholic
Guéchot et al. (43)	2000	91	91	Serum hyaluronan Albumin Bilirubin Platelets ALP INR CP score	Retrospective cohort	Non-alcoholic
Guha et al. (44)	2019	379	379	ALBI score MELD score	Prospective cohort	Non-alcoholic
Hartl et al. (45)	2021	663	307	Renin ProBNP Copeptin	Retrospective cohort	Non-alcoholic
Hsu et al. (46)	2021	3,722	3,722	ALBI-FIB4 score MELD score	Retrospective cohort	Non-alcoholic
Jain et al. (23)	2021	168	168	Thick fibrous septa Albumin Bilirubin Platelets INR Creatinine MELD score CP score	Retrospective cohort	Non-alcoholic
Jindal et al. (47)	2022	626	626	Liver stiffness Albumin	Prospective cohort	Non-alcoholic
Jindal et al. (48)	2020	741	741	HVPG	Retrospective cohort	Non-alcoholic
Kim et al. (28)	2012	217	217	Liver stiffness Platelets	Prospective cohort	Non-alcoholic
Kondo et al. (20)	2016	236	110	Portal haemodynamics Albumin AST/ALT MELD score	Retrospective cohort	Non-alcoholic
Kwon et al. (49)	2021	1,027	1,027	Liver-to-spleen volume ratio MELD score CP score	Retrospective cohort	Non-alcoholic
Merchante et al. (50)	2012	239	239	Liver stiffness MELD score CP score	Prospective cohort	Non-alcoholic
Merchante et al. (51)	2015	275	275	Liver stiffness	Prospective cohort	Non-alcoholic
Navadurong et al. (52)	2023	123	123	ALBI	Retrospective cohort	Non-alcoholic
Qamar et al. (53)	2009	213	213	Hematologic indices	RCT	Non-alcoholic
Piecha et al. (29)	2025	20	20	Epithelial cell death markers	Prospective cohort	Alcoholic
Rincón et al. (27)	2013	145	145	HVPG Albumin Platelets MELD score	Retrospective cohort	Non-alcoholic
Ripoll et al. (18)	2007	213	213	HVPG Albumin Platelets AST/ALT MELD score CP score	RCT	Non-alcoholic
Saeki et al. (54)	2023	148	148	Insulin-like growth factor 1 Albumin	Retrospective cohort	Non-alcoholic
Schneider et al. (55)	2022	6,049	6,049	EPOD score Albumin Bilirubin Platelets	Retrospective cohort	Non-alcoholic
Schwarzer et al. (56)	2020	194	194	VITRO score MELD score	Prospective cohort	Non-alcoholic
Siramolpiwat et al. (57)	2021	152	152	LFI Albumin Bilirubin MELD score CP score	Prospective cohort	Non-alcoholic
Smith et al. (58)	2017	830	326	Liver surface nodularity	Retrospective cohort	Non-alcoholic
Tapper et al. (59)	2020	274	111	Subcutaneous fat density MELD score	Prospective cohort	Non-alcoholic
Tornai et al. (60)	2021	244	101	Serum ferritin	Retrospective cohort	Non-alcoholic
Yang et al. (61)	2021	292	197	T2 mapping Albumin MELD score	Retrospective cohort	Non-alcoholic
Yoo et al. (62)	2024	101	101	CPA INR APRI	Retrospective cohort	Non-alcoholic
Yu et al. (63)	2022	689	689	Spleen volume	Retrospective cohort	Non-alcoholic
Zarski et al. (64)	2020	219	219	Liver stiffness	Retrospective case–control	Non-alcoholic

ABIDE, albumin-bilirubin-international normalized ratio–derived estimate; ALBI, albumin-bilirubin score; ALBI-FIB4, combined score using ALBI and fibrosis-4 index for liver fibrosis; ALP, alkaline phosphatase; ALT, alanine aminotransferase; APRI, AST to platelet ratio index; AST, aspartate aminotransferase; BMI, body mass index; CCL-21, chemokine ligand 21; CPA, collagen proportionate area; ELF score, enhanced liver fibrosis score; EPOD score, emerging prognostic outcome determinant score; FF, fibrosis fraction; GGT, gamma-glutamyl transferase; gp130, glycoprotein 130; HA, hyaluronic acid; HVPG, hepatic venous pressure gradient; IEV, interferon-gamma inducible protein (pro-inflammatory cytokine); IGFBP-7, insulin-like growth factor binding protein 7; IL-6, interleukin-6; INR, international normalized ratio; LFI, liver frailty index; M30, apoptosis marker; M65, total cytokeratin-18; MAP, mean arterial pressure; MELD, Model for end-stage liver disease; miR-181b-5p, microRNA associated with fibrosis progression; PIIINP, procollagen III N-terminal peptide; PLSec, plasma liver secretome panel; ProBNP, Pro–B-type natriuretic peptide; T2 mapping, MRI-based imaging technique using gadoxetic acid for liver fibrosis assessment; TIMP-1, tissue Inhibitor of metalloproteinases-1; VCAM-1, vascular cell adhesion molecule-1; VITRO score, von Willebrand factor and platelet-based score.

### 3.5 Biomarker frequency

Although multiple biomarkers were assessed across the included studies, only 11 biomarkers were selected for inclusion in the final pooled analysis. This selection was based on the completeness of reported data (i.e., availability of HRs and SEs), clinical relevance, and avoiding overlapping cohorts. Table 2 summarizes the most frequently investigated biomarkers. Biomarkers are grouped by category, and information is provided on the number of studies, total patient population (where applicable), predominant cirrhosis etiology (alcoholic vs. non-alcoholic), and study design.

### 3.6 Results of the meta-analysis

For analytical clarity, the 11 biomarkers selected for meta-analysis were grouped into six predefined categories: blood-based markers, liver stiffness, HVPG, imaging-based indicators, inflammatory markers, and miscellaneous. The selection of biomarkers for pooled analysis was informed by data completeness and cirrhosis etiology; biomarkers were included if sufficient and comparable data were available within alcoholic or non-alcoholic subgroups. In cases where multiple biomarkers were reported within a single study, only the most clinically relevant or prominently analyzed biomarker was retained. While not all studies are described in detail, this section highlights the most extensively studied and clinically significant biomarkers. The prognostic utility of serum biomarkers in liver cirrhosis has been widely investigated, particularly concerning their capacity to anticipate clinical decompensation. This meta-analysis evaluates three key laboratory parameters—serum albumin, total bilirubin, and platelet count—across etiologic subgroups of alcoholic and non-alcoholic cirrhosis, to assess their respective predictive performances (18).

Across eight studies involving non-alcoholic cirrhosis, low albumin levels were consistently associated with increased risk of decompensation. The pooled hazard ratio (HR) under the random-effects model was 0.40 [0.18, 0.90], indicating a protective effect of higher albumin concentrations. These findings are biologically plausible, as albumin reflects hepatic synthetic function and nutritional status. Notably, heterogeneity across studies was substantial (I^2^ ≈ 90%), suggesting population and methodological differences (17). In contrast, the alcoholic cirrhosis subgroup, represented by two studies, yielded a pooled HR of 0.94 [0.30, 2.99], with wide CIs and significant heterogeneity (*I*^2^ = 95.3%). This suggests a less reliable or more context-dependent prognostic role for albumin in alcohol-related liver disease, potentially influenced by malnutrition or systemic inflammation (19, 20). Subgroup comparison confirmed a statistically significant difference (*p* < 0.02), indicating that albumin is a stronger and more consistent prognostic marker in non-alcoholic cirrhosis.

Nevertheless, our meta-analysis of eight studies focusing on patients with non-alcoholic cirrhosis revealed a consistent association between reduced platelet count and increased risk of decompensation, yielding a pooled hazard ratio of 2.79 [2.63–2.96]. These findings underscore the potential utility of platelet count in risk-stratification models for non-ALD (21).

Bilirubin levels also showed a strong association with decompensation risk. In non-alcoholic cirrhosis (*n* = 4 studies), the pooled HR was 4.27 [2.93, 6.22], demonstrating a robust and statistically significant relationship. A single alcoholic cirrhosis study reported a comparable HR of 4.18 [3.39, 5.15]. Although subgroup comparison was limited by sample size, effect estimates suggest that bilirubin is a consistently strong predictor of decompensation across etiologies. Although the effect size is robust, the limited number of studies on alcoholic cirrhosis warrants cautious interpretation of the findings. Its role as a marker of hepatocellular excretion dysfunction supports this observation (22). The heterogeneity across studies remained moderate (*I*^2^ = 86%), reinforcing the robustness of bilirubin as a prognostic marker across different cirrhosis etiologies.

In contrast to albumin and bilirubin, platelet count did not demonstrate predictive significance in non-alcoholic cirrhosis. The pooled HR from seven studies was 1.01 [0.94, 1.08], with CIs overlapping unity, suggesting a null effect. Heterogeneity was moderate (*I*^2^ ≈ 51%).

However, one study on alcoholic cirrhosis reported an HR of 1.13 [1.03, 1.24], indicating a modest but significant increase in decompensation risk with lower platelet counts. This divergence may reflect the impact of portal hypertension or hypersplenism, which are more pronounced in alcohol-related liver disease.

**Figure 3:**
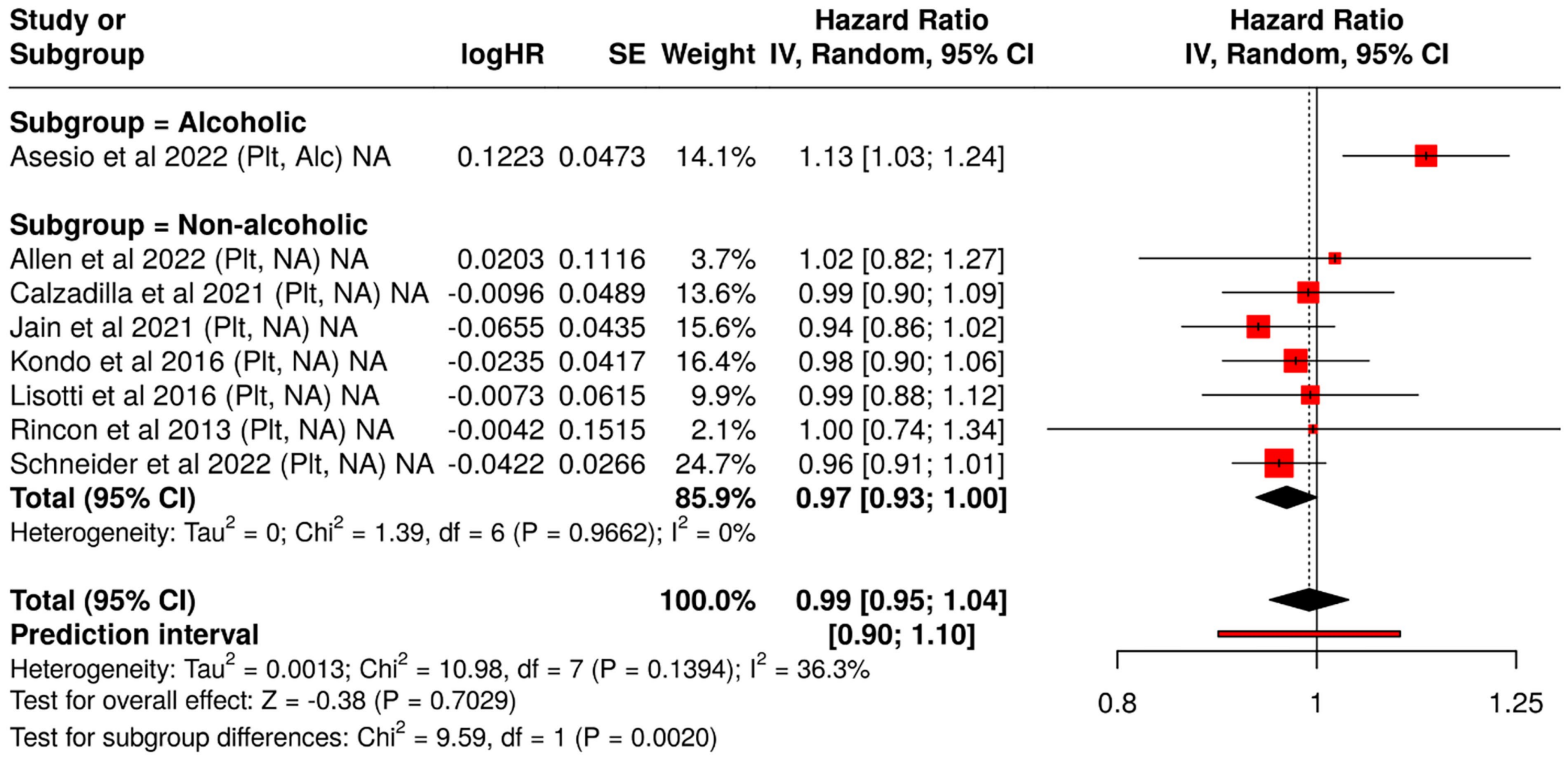
Forest plot analysis of blood-based biomarkers for predicting hepatic decompensation in compensated cirrhosis: subgroup comparison by etiology. This figure presents hazard ratios (HRs) and 95% confidence intervals for blood-based prognostic biomarkers associated with liver failure, stratified by cirrhosis etiology (alcoholic vs. non-alcoholic). The meta-analysis highlights differences in biomarker performance across subgroups, offering insight into etiology-specific predictive value.

Tests for subgroup differences confirmed statistical significance (*p* < 0.05), highlighting that the prognostic role of thrombocytopenia may be limited to alcoholic cirrhosis (Figure 3).

These findings emphasize the importance of etiology-specific interpretation of laboratory biomarkers in cirrhosis. Both albumin and bilirubin exhibit strong predictive value across etiologies, with greater consistency in non-alcoholic populations. In contrast, platelet count appears less informative overall, though it may retain some prognostic value in alcohol-associated liver disease.

Integration of these markers into risk stratification tools should consider disease etiology, pathophysiological context, and the interplay of systemic factors affecting biomarker levels.

As the understanding of cirrhosis pathophysiology evolves, attention has increasingly shifted toward dynamic and mechanistic biomarkers that extend beyond classical liver function tests. The current meta-analysis incorporated a series of novel and established indicators to evaluate their prognostic value in cirrhotic decompensation. This section highlights four such biomarkers: INR, K18, IL-6, and circulating extracellular vesicles (EVs)—each offering a distinct physiological lens (17, 19, 23).

A routine marker in liver disease monitoring, INR estimates coagulopathy via the activity of liver-synthesized clotting factors. In our analysis of three studies focusing on non-alcoholic cirrhosis, the data revealed considerable variability. While some individual studies reported significant associations with decompensation (e.g., HR = 6.89 [1.42, 33.45]), the combined estimate under a random-effects model yielded a wide and imprecise HR of 2.74 [0.20, 37.10], accompanied by substantial heterogeneity (*I*^2^ = 73.1%). The wide confidence interval reduces the precision of the estimate and suggests cautious interpretation.

Although elevated INR may indicate hepatic synthetic failure, its standalone prognostic strength appears inconsistent across non-alcoholic populations. It may be more effective when embedded within composite clinical scoring systems rather than used in isolation. K18 is a structural protein of hepatocytes, released into circulation upon apoptotic cell death, a common feature of alcoholic liver injury. A study of patients with alcoholic cirrhosis demonstrated a significant association between higher K18 levels and decompensation risk (HR = 1.77 [1.14, 2.75]) (24). K18 stands out as a biologically grounded marker of hepatocyte apoptosis. In alcoholic cirrhosis, its ability to reflect early cellular damage before overt clinical decline presents a compelling case for its integration into biomarker panels. As a key cytokine in the inflammatory cascade, IL-6 mediates hepatic immune activation and has been implicated in disease progression across liver disorders. In a 2025 study, elevated IL-6 in alcoholic cirrhosis was linked to a modestly increased hazard of decompensation (HR = 1.31 [1.00, 1.71]) (25). IL-6 represents the inflammatory axis of liver dysfunction. While the association observed here borders on significance, it suggests a contributory, not primary, role in decompensatory processes. Future studies with longitudinal designs may clarify its temporal relationship to clinical deterioration.

Extracellular vesicles, including exosomes and microvesicles, serve as messengers of intercellular stress signaling. Released from hepatocytes, immune, and endothelial cells, these vesicles carry molecular cargo that modulates inflammation and fibrogenesis. Data from provided striking evidence: elevated IEV levels in alcoholic cirrhosis patients were associated with a markedly increased risk of decompensation (HR = 5.09 [2.01, 12.86]) (24). EVs may represent a next-generation biomarker, integrating cellular injury, immune activation, and endothelial dysfunction into a measurable clinical signal. Their non-invasive detection and strong prognostic potential warrant further validation in multicenter cohorts.

**Figure 4:**
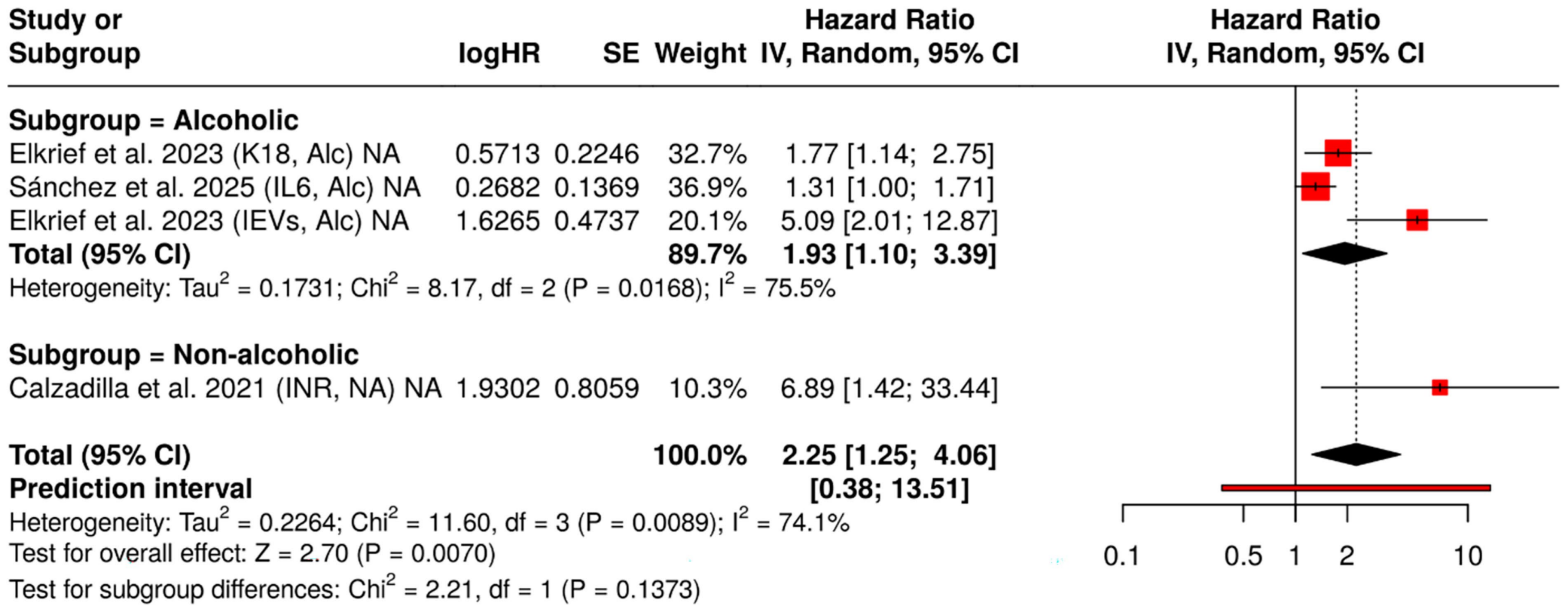
Forest plot of hazard ratios (HRs) for INR, keratin-18 (K18), interleukin-6 (IL-6), and extracellular vesicles (EVs), stratified by cirrhosis etiology. This figure displays HRs and 95% confidence intervals for selected prognostic biomarkers, comparing their predictive value for hepatic decompensation in patients with compensated cirrhosis of alcoholic and non-alcoholic origin. Data were synthesized using a meta-analysis approach to assess etiology-specific differences in biomarker performance.

Together, these emerging biomarkers highlight the layered complexity of cirrhotic decompensation, where hemodynamic, apoptotic, inflammatory, and molecular signals converge. Their roles are likely complementary rather than competing. While INR continues to reflect synthetic reserve, K18 and EVs may capture subtler shifts in cellular integrity and signaling. IL-6, albeit modest in effect, underscores the role of systemic inflammation (Figure 4).

In addition to circulating biomarkers and cytokines, several composite and hemodynamic parameters play a central role in predicting hepatic decompensation. This section synthesizes data from recent meta-analyses on three such markers — MELD score, LSM, and HVPG — stratified where applicable by cirrhosis etiology.

The MELD score, derived from serum bilirubin, INR, and creatinine, remains a cornerstone for prognostication in chronic liver disease. In our analysis of nine studies on non-alcoholic cirrhosis, MELD demonstrated a consistent association with decompensation risk (pooled HR = 1.13 [1.07, 1.20]). The effect persisted in alcoholic cirrhosis, with a higher point estimate (HR = 1.87 [1.12, 3.11]), suggesting more rapid deterioration in this group. Subgroup comparison confirmed a statistically significant difference in effect size (*p* = 0.0437) (24).

Model for end-stage liver disease is a validated, widely accessible predictor of decompensation, particularly relevant in clinical triage. Its predictive utility may be amplified in alcohol-related disease due to concurrent renal dysfunction or systemic inflammation.

LSM via transient elastography is a non-invasive surrogate for fibrosis and portal hypertension. Two studies included in the analysis revealed differing effect sizes across etiologies. In non-alcoholic cirrhosis a strong association with decompensation (HR = 1.26 [1.16, 1.36]), while in alcoholic cirrhosis (HR = 1.04 [1.02, 1.06]) (22, 26). The subgroup test was highly significant (*p* < 0.0001), reinforcing the etiology-dependent prognostic value of LSM. LSM is a valuable, non-invasive biomarker, particularly predictive in non-alcoholic cirrhosis. Its performance appears attenuated in alcoholic disease, possibly due to confounding inflammation or steatosis.

HVPG remains the invasive reference standard for assessing portal hypertension and confirmed its prognostic value in non-alcoholic cirrhosis, with a pooled HR of 1.13 [1.13, 1.13] per mmHg increase and no heterogeneity (*I*^2^ = 0%). HVPG offers unmatched precision as a predictor of decompensation (18, 27). Despite its invasive nature, its reliability and reproducibility justify its use in both clinical trials and high-risk patient populations.

Beyond functional and hemodynamic parameters, several imaging-based morphological markers have shown promise for predicting hepatic decompensation. Notably, spleen size and PVD serve as non-invasive surrogates for portal hypertension and splenoportal remodeling in advanced cirrhosis.

Splenomegaly reflects both increased portal pressure and hypersplenism, frequently accompanying compensated cirrhosis. In two studies conducted exclusively in non-alcoholic cirrhosis, spleen size was significantly associated with incident decompensation: The pooled hazard ratio was 5.79 [2.00, 16.80], suggesting a several-fold increase in risk (28, 29). Moderate heterogeneity (*I*^2^ = 65%) was observed, but both studies individually confirmed the association. Spleen enlargement is a clinically accessible and highly predictive morphological marker in non-alcoholic cirrhosis. Its diagnostic simplicity reinforces its role in surveillance imaging protocols.

Portal vein dilation is an established indicator of splanchnic vascular derangement. PVD was strongly predictive of hepatic decompensation in non-alcoholic cirrhosis, with a hazard ratio of 7.39 [4.90, 11.15] (29). PVD represents a robust and quantifiable predictor of decompensation. Its integration into ultrasound-based assessments enhances its clinical utility, especially in resource-limited settings.

**Figure 5:**
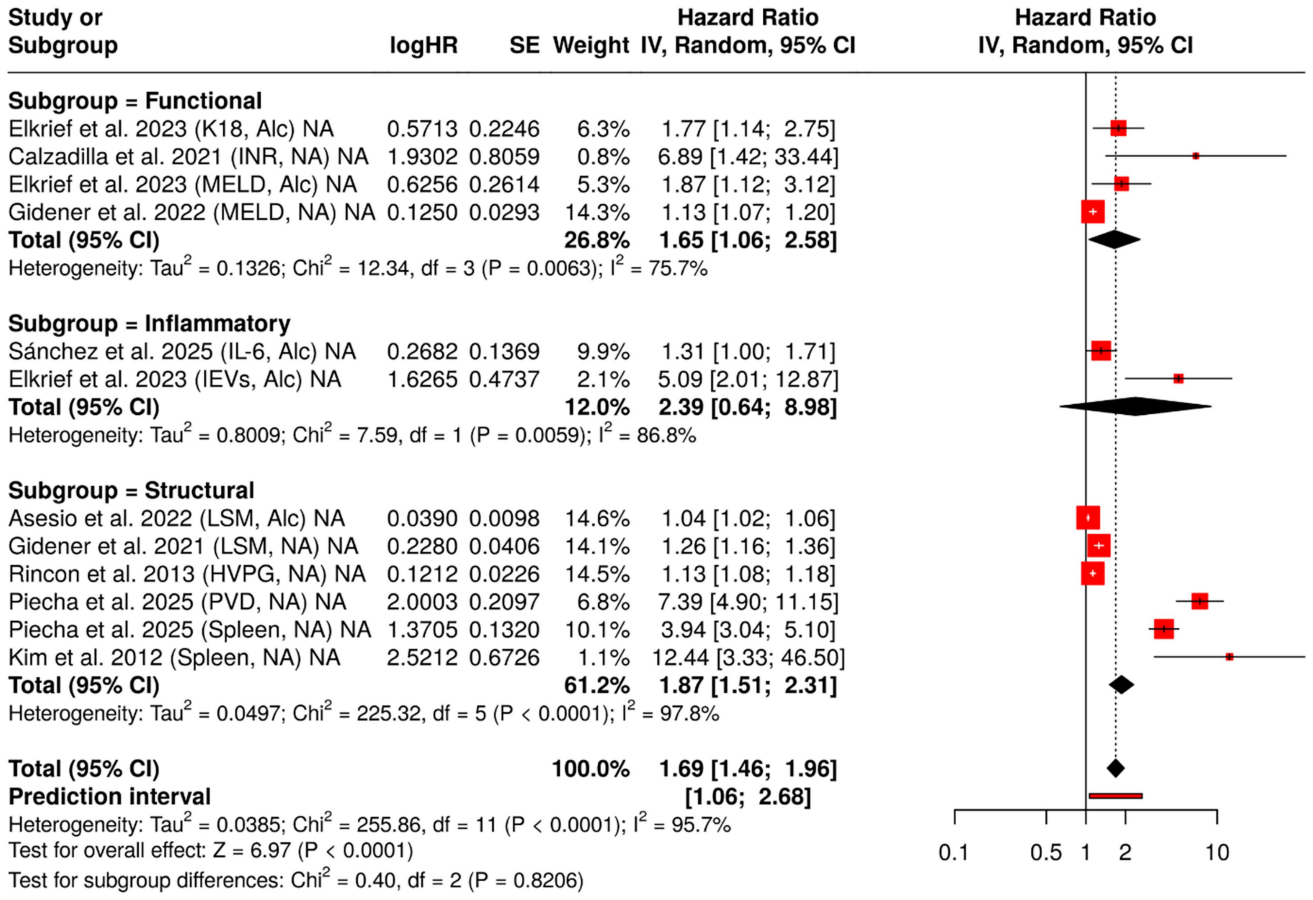
Forest plot of hazard ratios (HRs) for liver failure biomarkers, categorized by biological function. This figure presents HRs and 95% confidence intervals for 12 prognostic biomarkers associated with liver failure, grouped into three biological categories: inflammatory, functional, and structural. A random-effects model was applied to each group, and the plot illustrates the relative predictive value and heterogeneity (*I*^2^) for each biomarker. Data are derived from a meta-analysis evaluating prognostic biomarkers for predicting hepatic decompensation in patients with compensated cirrhosis, stratified by etiology (alcoholic vs. non-alcoholic).

Figure 5 presents a comparative analysis of the prognostic significance of 11 biomarkers for hepatic decompensation, grouped into three biological categories. Among the inflammatory biomarkers, EVs demonstrated the strongest correlation (HR = 5.09), whereas IL-6 showed a moderate but significant effect. Functional biomarkers such as the INR and the MELD consistently predicted outcomes in various etiologies (HR = 6.89 and HR = 1.87, respectively). Structural biomarkers, including PVD and spleen size, demonstrated the highest HRs (HR = 7.39 and 5.79), highlighting their importance in assessing portal hypertension. The figure emphasizes the diverse but complementary value of biomarkers from different biological domains.

**Figure 6:**
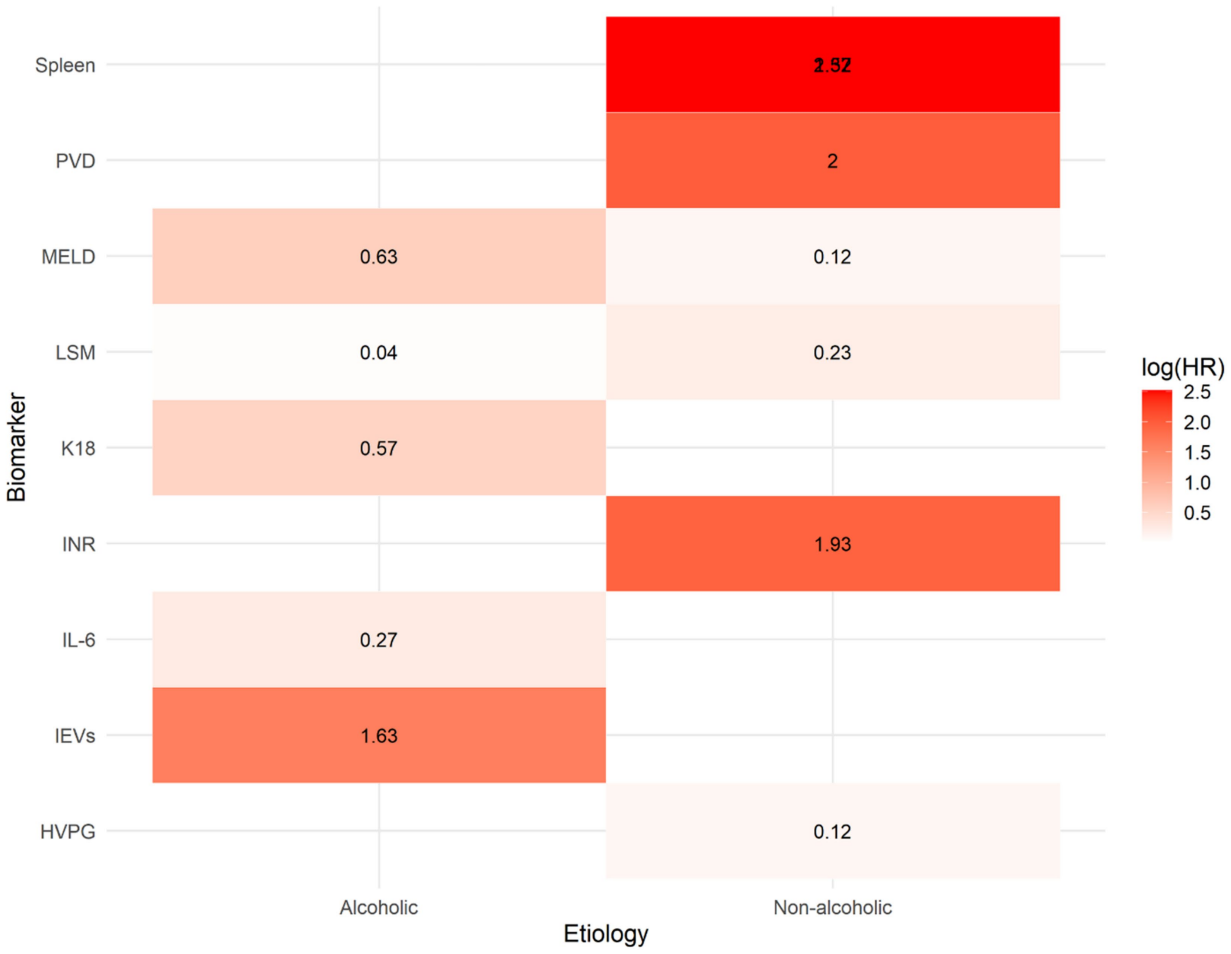
Heatmap of log-transformed hazard ratios (log[HR]) for prognostic biomarkers predicting hepatic decompensation in patients with compensated cirrhosis, stratified by etiology (alcoholic vs. non-alcoholic). Each cell represents the log-transformed hazard ratio derived from a meta-analysis of the association between a specific biomarker and liver failure. Warmer colors (red) indicate stronger positive associations (higher HR), while cooler colors (blue) reflect weaker associations. The heatmap highlights that structural biomarkers such as portal vein diameter (PVD) and spleen size, along with inflammatory markers, have the highest predictive value, particularly in cases of non-alcoholic cirrhosis.

Figure 6 illustrates a heatmap of the log-transformed HRs (log (HR)), which summarize the strength of the relationship between 12 biomarkers and the risk of hepatic decompensation, stratified by underlying cause of cirrhosis (alcohol-related versus non-alcohol related). The biomarkers were arranged based on their biological categories, including inflammatory mediators such as IL-6 and EVs, functional indices such as INR, MELD and K18, and structural measurements such as spleen size, PVD and HVPG.

The warm colors (red hues) indicate a strong association with decompensation [high log(HR)] while the cool colors (blue tones) suggest a weak or no effect. The heat map reveals several significant patterns: structural markers such as PVD and spleen size exhibit the highest log(HR values), particularly in patients with non-alcohol related cirrhosis.

Inflammatory indicators, particularly EVs, exhibit strong predictive capabilities in individuals with alcoholic cirrhosis. In contrast, IL-6 and K18 demonstrate modest, yet biologically plausible correlations. Functional markers such as the MELD and INR consistently demonstrate positive correlations across groups, although these correlations are more pronounced in non-alcoholic cases. This analysis supports the notion that biomarkers have different prognostic significance depending on the underlying cause of cirrhosis and emphasizes the need for personalized risk assessment models.

### 3.7 Reporting biases

**Figure 7:**
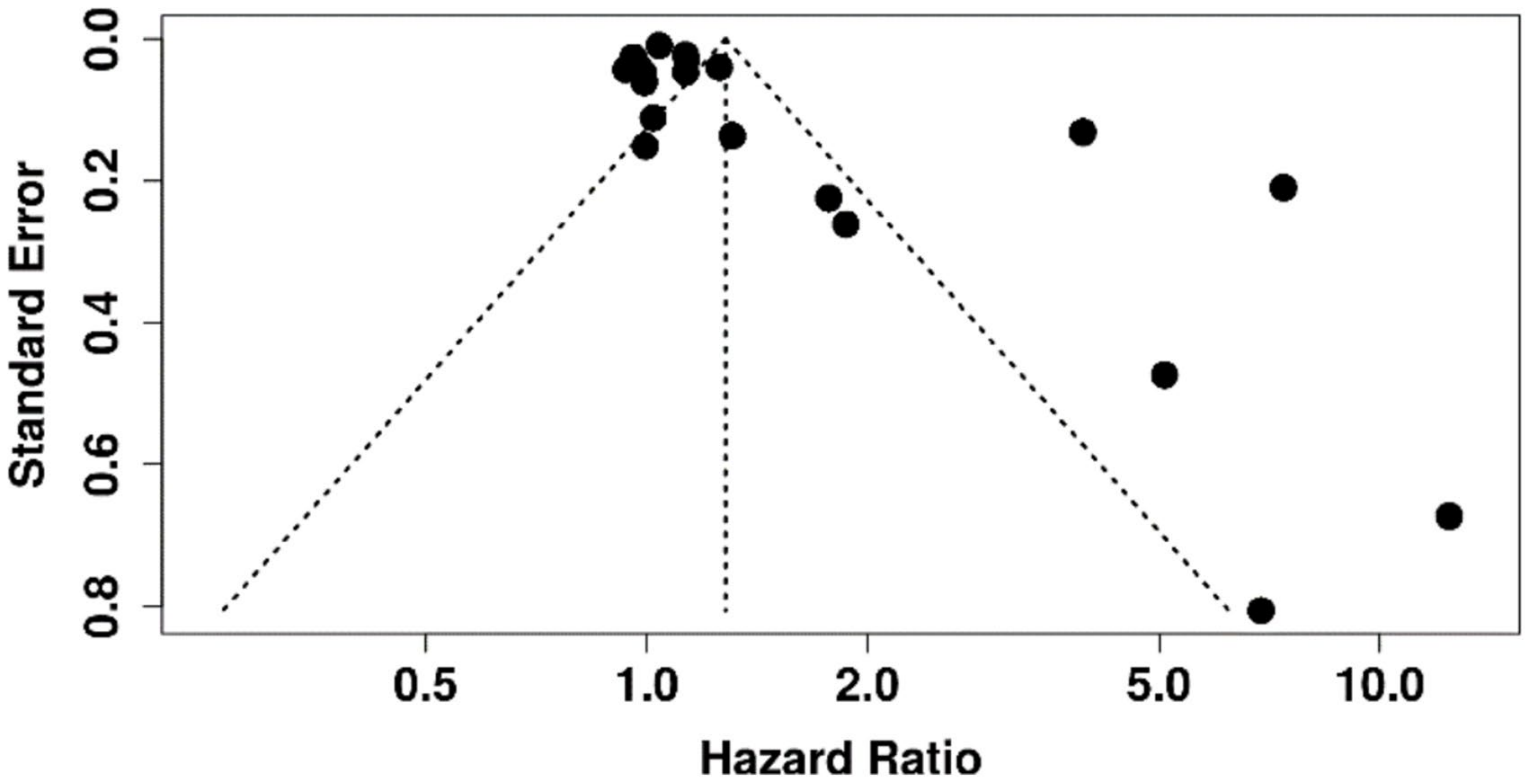
Funnel plot assessing publication bias in meta-analysis of prognostic biomarkers for predicting decompensation in alcoholic and non-alcoholic patients with compensated cirrhosis.

Funnel plots for biomarkers with ≥10 studies (e.g., albumin and bilirubin) showed no clear asymmetry, suggesting low risk of publication bias. Egger’s test was non-significant (*p* > 0.05). Selective reporting could not be fully assessed due to limited protocol availability (Figure 7).

## 4 Discussion

This comprehensive analysis examined both well-established and novel indicators to predict hepatic deterioration in individuals with compensated cirrhosis. The participants were divided into two groups based on their underlying cause – alcoholic or non-alcoholic – and the data from 66 studies were combined to assess the clinical significance and discriminative power of these markers.

Traditional scores, such as the MELD and the INR, showed moderate predictive value. The INR was the most significant traditional marker, especially in non-alcoholic patients, consistent with the findings from large meta-analyses (30). Our findings are consistent with those reported in a recent meta-analysis by Gananandan et al. (30), which highlighted the moderate predictive capacity of conventional scoring systems like MELD and ALBI. However, our study advances this by incorporating novel biomarkers such as EVs and K18 and stratifying results by cirrhosis etiology, an approach not taken in previous reviews.

The parameters of portal hypertension, particularly the PVD and the size of the spleen, demonstrate the highest risk ratios (~5–7) with significant predictive value for non-alcoholic patients. This indicates the potential of imaging-based classification to identify individuals at risk in early stages. An important consideration is that TIPS procedures, which reduce portal pressure and may normalize PVD, were not consistently reported in the included studies. This absence limits our ability to control for post-TIPS confounding, and thus, the prognostic significance of PVD must be interpreted with caution.

Among the novel biomarkers, EVs originating from hepatocytes and K18 have shown great promise in predicting the prognosis of alcoholic cirrhosis. The HRs for these biomarkers were approximately 5.09 and 1.77, respectively. These findings align with the large-scale prospective study by Elkrief et al. (24), which reported that combining hepatocyte-derived biomarkers such as keratin 18 and extracellular vesicles with the MELD score significantly improved prediction of liver-related events in patients with compensated alcoholic cirrhosis (HR for EVs = 5.09; for K18 = 1.77) compared to using MELD alone. Moreover, it was discovered that IL-6 was a reliable predictor across various causes, indicating that systemic inflammation is a crucial factor in the process of decompensation (67). This finding aligns with the results where IL-6 outperformed MELD in predicting short-term mortality in patients with decompensated liver disease. The predictive value of LSM and HVPG further underscores the importance of monitoring structural and hemodynamic changes. HVPG demonstrated a strong and consistent correlation with mortality, with a HR of approximately 1.13 and minimal variability.

Heatmap visualizations and subgroup forest plots of log (HR) values helped to identify key patterns in biomarkers based on the underlying cause of liver disease. However, there was some variation in certain areas, such as confounding and attrition. Limitations of the review process include the restriction to English-language peer-reviewed articles, potentially missing relevant gray literature, and the lack of contact with study authors for missing data. High heterogeneity (I^2^ > 50%) may reflect variability in study populations and biomarker measurement methods.

## 5 Conclusion

This meta-analysis highlights the importance of tailoring prognostic assessment in compensated cirrhosis based on disease etiology. While conventional markers like MELD and INR retain moderate predictive utility, their performance is enhanced when complemented by etiology-specific indicators. In non-alcoholic cirrhosis, structural metrics such as spleen size and PVD emerged as strong predictors of decompensation, emphasizing the relevance of imaging and hemodynamic evaluation. In contrast, alcoholic cirrhosis was more accurately predicted by hepatocyte-derived biomarkers, including EVs and K18, both demonstrating substantial HRs. Additionally, IL-6 consistently predicted outcomes across both subtypes, underscoring the central role of systemic inflammation in cirrhotic progression. Collectively, these findings support the integration of structural, functional, and inflammatory biomarkers into stratified prognostic frameworks. The results lay a foundation for the development of non-invasive, individualized risk models and highlight the need for prospective validation using standardized methodologies.

## Data Availability

The data that support the findings of this study are available from the corresponding author, [A.T.], upon reasonable request.
